# Urine biomarkers for the prediction of mortality in COVID-19 hospitalized patients

**DOI:** 10.1038/s41598-021-90610-y

**Published:** 2021-05-27

**Authors:** Daniel Morell-Garcia, David Ramos-Chavarino, Josep M. Bauça, Paula Argente del Castillo, Maria Antonieta Ballesteros-Vizoso, Luis García de Guadiana-Romualdo, Cristina Gómez-Cobo, J. Albert Pou, Rocío Amezaga-Menéndez, Alberto Alonso-Fernández, Isabel Llompart, Ana García-Raja

**Affiliations:** 1 Laboratory Medicine Department, Hospital Universitari Son Espases, Ctra. Valldemossa 79, mòdul 0-J., 07120 Palma de Mallorca, Spain; 2 Institut d’Investigació Sanitària de Les Illes Balears (IdISBa), Palma de Mallorca, Spain; 3 Laboratory Medicine Department, Hospital Universitario Santa Lucía, Cartagena, Spain; 4 Internal Medicine Department, Hospital Universitari Son Espases, Palma de Mallorca, Spain; 5 Intensive Care Unit, Hospital Universitari Son Espases, Palma de Mallorca, Spain; 6 Respiratory Medicine Department, Hospital Universitari Son Espases, Palma de Mallorca, Spain

**Keywords:** Biochemistry, Microbiology, Biomarkers, Nephrology, Risk factors, Urology

## Abstract

Risk factors associated with severity and mortality attributable to COVID-19 have been reported in different cohorts, highlighting the occurrence of acute kidney injury (AKI) in 25% of them. Among other, SARS-CoV-2 targets renal tubular cells and can cause acute renal damage. The aim of the present study was to evaluate the usefulness of urinary parameters in predicting intensive care unit (ICU) admission, mortality and development of AKI in hospitalized patients with COVID-19. Retrospective observational study, in a tertiary care hospital, between March 1st and April 19th, 2020. We recruited adult patients admitted consecutively and positive for SARS-CoV-2. Urinary and serum biomarkers were correlated with clinical outcomes (AKI, ICU admission, hospital discharge and in-hospital mortality) and evaluated using a logistic regression model and ROC curves. A total of 199 COVID-19 hospitalized patients were included. In AKI, the logistic regression model with a highest area under the curve (AUC) was reached by the combination of urine blood and previous chronic kidney disease, with an AUC of 0.676 (95%CI 0.512–0.840; *p* = 0.023); urine specific weight, sodium and albumin in serum, with an AUC of 0.837 (95% CI 0.766–0.909; *p* < 0.001) for ICU admission; and age, urine blood and lactate dehydrogenase levels in serum, with an AUC of 0.923 (95%CI 0.866–0.979; *p* < 0.001) for mortality prediction. For hospitalized patients with COVID-19, renal involvement and early alterations of urinary and serum parameters are useful as prognostic factors of AKI, the need for ICU admission and death.

## Introduction

Coronavirus disease (COVID-19), produced by the new RNA virus called SARS-CoV-2, was discovered in late 2019 in China^1,2^. COVID-19 is characterized by a wide spectrum of symptoms, ranging from an asymptomatic state to an acute respiratory distress syndrome in the adult (ARDS), being the acute respiratory tract infection the main affection, possibly progressing to a severe pneumonia^3–5^.

In different retrospective cohorts, several severity and mortality-associated risk factors have been reported^6–8^, with acute kidney injury (AKI) being described in up to 25% of patients^6,9^. The onset of AKI in individuals with ARDS implies a significant increase in ARDS-associated mortality rates^10^. Different studies highlight the importance of an elevation of inflammatory mediators as the pathogenic basis for the development of AKI, produced by an endothelial dysfunction, microangiopathy and tubular damage^11^.

Patients with severe COVID-19 may present with a status of systemic hyperinflammation or cytokine storm^12^. In addition, SARS-CoV-2 uses the angiotensin-converting enzyme receptor 2 (ACE2) as target for cell invasion, which is also present on the surface of renal tubular cells^13^. AKI onset has been described on the 9 days after hospital admission due to COVID-19, and associated to factors such as age, infection severity, cardiorenal syndrome and diabetes^6,9^.

Renal complications are common in individuals with comorbidities that predispose to kidney disease, being difficult to evaluate by means of serum creatinine measurements. To date, some previous studies have reported the usefulness of urine strip biochemical results on the prediction of severity of COVID-19 patients^14^.

In this line, the aim of this study was to assess the urinary parameters that, in combination with other laboratory tests in serum, demographics variables and comorbidities, could be useful for predicting the development of AKI, requirement of ICU admission and in-hospital mortality, in hospitalized patients with COVID-19.

## Methods

### Study design and participants

This is a retrospective observational study performed in a tertiary care hospital between March 1st and April 19th, 2020. Patients above 18 years old were consecutively selected through a search on the laboratory information system, including only patients with a positive result on the real-time reverse transcriptase–polymerase chain reaction (RT-PCR) due to a clinical suspicion of COVID-19 and a urine dipstick study requested. Individuals without hospital admission were excluded, alongside with those with hospital admission and a confirmed urinary tract infection (UTI), defined as a positive urine culture.

The prevalence of AKI^15^ and/or admission to the Intensive Care Unit (ICU) was recorded, and patients were monitored until ICU discharge, hospital discharge or patient’s death. Patients who at the time of retrospective recruitment did not have a final event and remained in ICU were excluded.

### Clinical and biochemical records

Epidemiologic, demographic, clinical and biochemical variables were analyzed for all individuals.

The recorded data included time from positive RT-PCR to urinalysis, time from urinalysis to clinical endpoints (AKI, ICU admission, discharge or death) and presence of AKI risk factors coded in the medical record (history of heart failure, obesity, diabetes, hypertension and chronic kidney disease (CKD)).

Laboratory confirmation of SARS-CoV-2 infection was defined as a positive result of RT-PCR assay on samples collected on nasal and pharyngeal swabs^16^. Laboratory data included a complete urinalysis and urine sediment, which were performed on an automatic biochemical and flow cytometer sediment analyzer (Sysmex series UC-3500, UF-5000 and UD-10; Sysmex, Japan; Urine Dipstick MEDITAPE UC-11A and UC-9A), respectively. Approximately 10 mL of midstream urine samples, or collected from urinary catheter in critical patients, were obtained from individuals, and all of them were analyzed within 2 h. To confirm doubtful formed elements in urine, samples were centrifuged 5 min at 1000 × g and checked by optical microscopy by a senior consultant.

Blood was also collected from patients in the Emergency Room or during hospitalization on the same day of urinalysis and onwards. The following tests were performed: complete blood count (Cell-Dyn Sapphire platform, Abbott Diagnostics, US), D-dimer (HemosIL D-Dimer HS, DDU, ACL-TOP 700, Instrumentation Laboratory, US), serum biomarkers: liver enzymes, C-reactive protein (CRP), lactate dehydrogenase (LDH), creatinine phosphokinase (CK), ferritin, urea, albumin, creatinine, potassium, sodium and chloride (Architect ci16200 platform, Abbott Diagnostics, US).

### Definitions

For the diagnosis and staging of AKI, the 2012 Kidney Disease: Improving Global Outcomes (KDIGO) definition was used^15^. Individuals were screened considering the AKI 2012 KDIGO definition as major criterion. For individuals without any previous serum creatinine result, a 7-day follow-up was performed for the establishment of the AKI onset during the episode. Serum creatinine concentration upon admission was considered as baseline.

Chronic kidney disease (CKD) was defined as an estimated glomerular filtration rate (eGFR) < 60 ml/min per 1.73 m^2^ or a urine albumin-creatinine ratio ≥ 30 µg/mg at least 3 months before admission^17^. Presence of proteinuria and hematuria were defined as more than trace albumin or blood on urine dipstick tests (> 0.3 g/L and > 0.06 mg/dL, respectively). The cut-off values for the other parameters included in the urine dipstick analysis were the following: urobilinogen (> 34.0 µmol/L), glucose (> 2.8 mmol/L), ketones (> 0.93 mmol/L) and bilirubin (> 8.6 µmol/L). Obesity was defined as a body mass index over than 30 kg/m^2^. AKI recovery was defined as a complete recovery of the kidney function, with a serum creatinine decreasing below threshold or to the baseline.

### Statistical analysis

The sample size was calculated for the smallest of the differences described in the bibliography for the three events, the smallest being the need for admission to the ICU with a percentage difference of 16.4 points^18^ to local data, for a statistical power of 90% and an alpha type error of 0.05, 183 subjects are required in total for the present study. This criterion was used because it was the data we could most reliably approximate, since both deaths were strongly age-dependent and included percentage ranges ranging from values below 1% to age groups with more than 20% mortality. On the other hand, the incidence of AKI in these patients has also been subject to controversy, and the series' ranges vary widely. The sample size calculation and given the retrospective nature of the analysis, we included 61 COVID-19 patients by group, given the study of the three endpoints (mortality, ICU admission and AKI), results in total of 183 patients were sufficient to detect the a priori differences expected from the literature. Regard to group subanalyses, if we consider a subsample group vs. the total sample with a statistical power of 90%, an alpha error of 5% and a beta error of 10%, for an expected increase in incidence of 16.4%, the number of events we need in each subgroup is 3 patients presenting the event death, ICU admission and AKI.

Descriptive statistics included frequency analysis (percentages) for categorical variables and means and standard deviation or medians and interquartile ranges amplitude (IQR = p75-p25) for continuous variables. Sample normality was assessed through the Kolmogorov–Smirnov’s test and comparisons were determined by the Student’s t or Mann–Whitney’s U tests for continuous variables, as appropriate, and by the use of the Chi squared test or Fisher’s exact test for categorical variables, after verifying the variances using the Levene´s test. A binary and multiple logistic regression was performed, and stepwise regression with forward selection method (best Fisher’s test sequence) was used for predicting variables. Odds ratios (ORs) were calculated for each significant variable and receiver-operating characteristics (ROC) curve analyses were performed with the different cut-off values, selecting those with the best Chi squared value, of the significant parameters of the logistic regression model for the discrimination of COVID-19 clinical endpoints. The differences were considered statistically significant when the alpha type error was less than 0.05. SPSS v.24 software was used for data analysis (IBM Corporation, US).

### Ethics approval and consent to participate

The Local Ethics Committee (Comitè d’Ètica de la Investigació de les Illes Balears) approved the study (IB4258/20PI) and all individuals or their legal representatives gave their informed consent. Because of Spanish laws, the research team cannot share the full database used for the current paper. Moreover, because COVID-19 admitted patients to our hospital was limited, data could contains potentially identifying or sensitive patient information. However, other researchers who meet the criteria for access to confidential data, may request to gain access to the minimal data set underlying the results under request at the Ethics Committee (contact via https://www.caib.es/sites/comiteetic/es/portada44578/?campa=yes), (e-mail address: ceic_ib@caib.es).

### Human investigations

We certify that all protocols and methods are carried out in accordance with relevant guidelines and regulations.

## Results

### COVID-19 screening population

**Figure 1:**
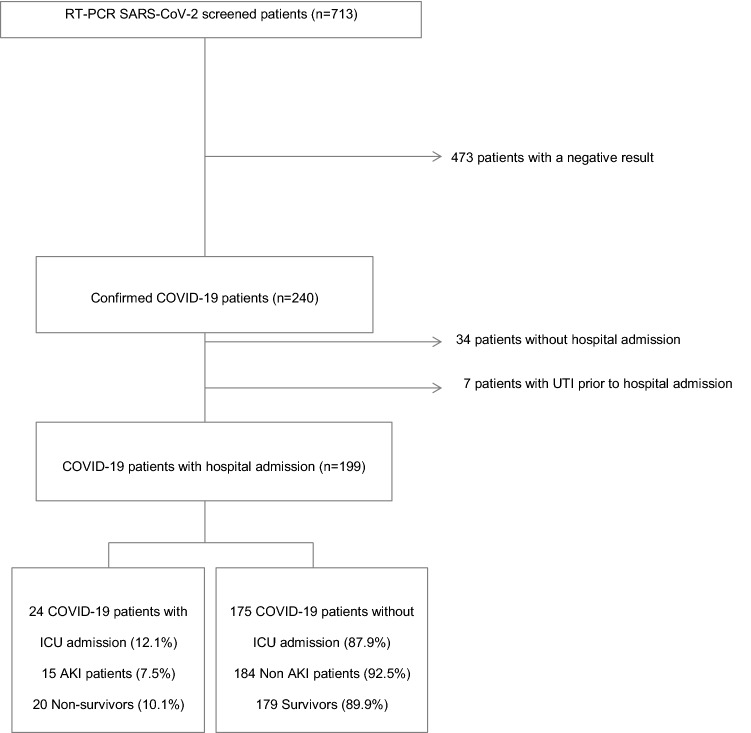
Flow chart study.

During the study period, a total of 713 patients with a RT-PCR analysis for SARS-CoV-2 were included, of which 240 (33.7%) had a positive result (Fig. 1).

A total of 199 (82.9%) individuals with a positive result required hospital admission. Clinical data and biochemical parameters of COVID-19 hospitalized patients are compared in Table 1, has been described with differentiated genders, as gender is an independent variable associated with the risk of presenting the three endpoints described. Median time from a positive RT-PCR result to urinalysis was 0.32 days (IQR: 2.16).

**Table 1:** Clinical data and biochemical parameters of COVID-19 hospitalized patients.

COVID-19 hospitalized patients	Total	Men	Women	p*
N	199 (100%)	121 (60.8%)	78 (39.2%)	< 0.001
Age (years)	58.9 ± 16.2	59.0 ± 16.5	58.8 ± 15.9	0.908
DM (n, %)	27 (13.6)	21 (17.4)	6 (7.7)	0.054
HT (n, %)	75 (37.7)	51 (42.2)	24 (30.8)	0.105
Obesity (n, %)	19 (9.5)	13 (10.7)	6 (7.7)	0.472
CKD (n, %)	15 (7.5)	14 (11.6)	1 (1.3)	0.002
HF (n, %)	1 (0.5)	0 (0.0)	1 (1.3)	0.317
ICU admission (n, %)	24 (12.1)	21 (17.4)	3 (3.9)	0.003
AKI (n, %)	15 (7.5)	11 (9.1)	4 (5.1)	0.267
KDIGO Stage 1 (n, %)	10 (66.7)	7 (63.6)	3 (75.0)	0.682
KDIGO Stage 2 (n, %)	2 (13.3)	2 (18.2)	0	0.358
KDIGO Stage 3 (n, %)	3 (20.0)	2 (18.2)	1 (25.0)	0.772
Non-survivors (n, %)	20 (10.1)	17 (14.1)	3 (3.9)	0.014
**Urinalysis parameters**				
pH	5.75 (1.0)	5.5 (0.5)	6.0 (1.5)	0.001
Specific weight	1.021 ± 0.094	1.023 ± 0.010	1.018 ± 0.084	< 0.001
Urobilinogen presence (n, %)	27/199 (13.6)	18/121 (14.9)	9/78 (11.5)	0.685
Blood presence (n, %)	35/199 (17.6)	22/121 (18.2)	13/78 (16.7)	0.576
Protein presence (n, %)	100/199 (50.3)	76/121 (62.8)	24/78 (30.8)	< 0.001
Glucose presence (n, %)	14/199 (7.0)	12/121 (9.9)	2/78 (2.6)	0.374
Ketones presence (n, %)	40/199 (20.1)	29/121 (24.0)	11/78 (14.1)	0.152
Bilirubin presence (n, %)	4/199 (2.0)	4/121 (3.3)	0/78 (0.0)	0.157
Nitrite positive (n, %)	7/199 (3.5)	3/121 (2.5)	4/78 (5.1)	0.436
**Sediment parameters**				
RBC (cells/uL)	23.2 (37.5)	19.8 (34.3)	26.6 (40.6)	0.181
WBC (cells/uL)	13.7 (42.4)	5.1 (7.8)	22.2 (77.0)	0.172
Bacteria (CFU/uL)	92.3 (228.3)	10.6 (39.8)	173.9 (416.7)	0.068
Epithelial cells (cells/uL)	5.9 (15.1)	1.4 (3.0)	10.4 (27.3)	0.014
Non-squamous cells (cells/uL)	6.6 (13.4)	6.3 (12.8)	6.9 (13.9)	0.382
Transitional cells (cells/uL)	0.3 (0.5)	0.1 (0.2)	0.4 (0.7)	0.006
Renal tubular cells (cells/uL)	6.4 (13.3)	6.1 (12.7)	6.6 (13.9)	0.438
Casts (n, %)	116/199 (58.3)	73/121 (60.3)	43/78 (55.1)	0.465
Hyaline casts (cast/uL)	0.3 (0.8)	0.3 (0.8)	0.3 (0.7)	0.605
Granular casts (cast/uL)	0.1 (0.6)	0.2 (0.7)	0.1 (0.4)	0.750
Mucus (uL)	0.1 (0.3)	0.1 (0.4)	0.1 (0.2)	0.848
**Blood parameters**				
Hemoglobin (g/dL)	14.2 ± 1.5	14.8 ± 1.6	13.6 ± 1.4	< 0.001
Lymphocytes (10^3^cells/uL)	1.2 (0.6)	1.1 (0.6)	1.3 (0.6)	0.723
Platelets (10^3^cells/uL)	201 (94)	182 (87)	219 (102)	0.004
D-Dimer (ng/mL)	244 (249)	259 (297)	229 (201)	0.081
Creatinine (mg/dL)	0.82 (0.27)	0.92 (0.40)	0.72 (0.14)	< 0.001
Urea (mg/dL)	32 (18)	36 (24)	28 (13)	< 0.001
Sodium (mmol/L)	138 (5)	137 (5)	138 (4)	0.168
Potassium (mmol/L)	4.0 (0.6)	4.0 (0.6)	4.0 (0.6)	0.147
Chloride (mmol/L)	104 (5)	105 (5)	103 (5)	0.082
Urate (mg/dL)	4.3 (2.5)	4.6 (2.8)	3.9 (2.2)	0.035
Albumin (g/L)	36.4 ± 3.9	36.2 ± 4.1	36.6 ± 3.8	0.460
AST (U/L)	29 (25)	34 (30)	24 (19)	0.003
LDH (U/L)	311 (145)	328 (163)	293 (128)	0.006
CK (U/L)	69 (81)	99 (129)	39 (34)	< 0.001
Ferritin (ng/mL)	541 (886)	817 (1,399)	264 (373)	0.038
CRP (mg/dL)	6.64 (9.90)	8.85 (10.62)	4.42 (9.18)	0.011

(*) men-women comparison; Quantitative variables were described by mean ± standard deviation or median (IQR); DM, Diabetes; HT, Hypertension; CKD, Chronic kidney disease; HF, heart failure; ICU, Intensive Care Unit; AKI, Acute Kidney Injury; RBC, Red Blood Cells; WBC, White Blood Cells; CFU, colony forming units; AST, aspartate aminotransferase; LDH, lactate dehydrogenase; CK, creatine kinase; CRP, C-reactive protein; KDIGO, Kidney Disease Improving Global Outcomes.

### Clinical endpoints for hospitalized patients with COVID-19

#### Intensive care unit admission

**Figure 2:**
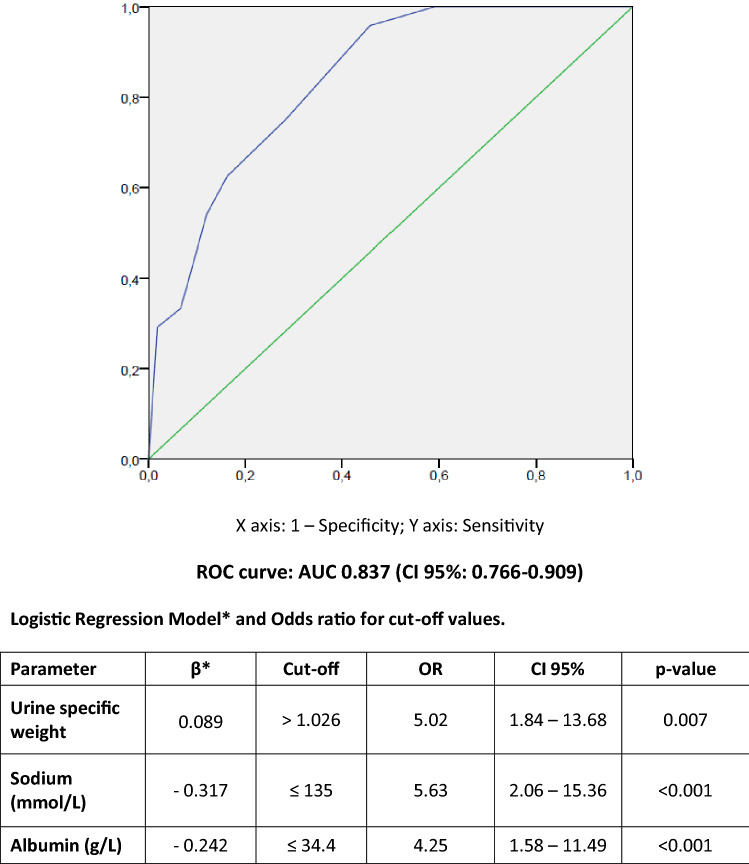
Cut-off values and ROC curve of urine specific weight, sodium and albumin in serum for COVID-19 critically prediction.

Twenty-four patients needed ICU admission, which represents 12.1% of the number of hospitalized patients. Clinical variables and biochemical results for critical and non-critical patients are shown in Table 2. The ROC curve analyses performed on the different significant clinical variables and biomarkers revealed that the highest area under the curve (AUC) was reached by a combination of urine specific weight, sodium and albumin in serum, with an AUC of 0.837 (95% CI 0.766–0.909; *p* < 0.001). Different cut-off values of specific weight, sodium and albumin in serum were tested in order to evaluate the OR for all of them. Figure 2 summarizes those cut-off values for the prediction for this endpoint and the OR and ROC curve obtained thereof. Median time from urinalysis result to ICU admission was 2.17 days (IQR: 2.91).

**Table 2:** Clinical data and biochemical results for critical and non-critical COVID-19 patients.

Hospitalized patients	ICU	Non-ICU	*p*
N, %	24 (12.1%)	175 (87.9%)	< 0.001
Age (years)	59.7 ± 12.1	58.8 ± 16.7	0.653
DM (n, %)	6/24 (25.0)	21/175 (12.0)	0.018
HT (n, %)	11/24 (45.8)	64/175 (36.8)	0.379
Obesity (n, %)	6/24 (25.0)	13/175 (7.4)	0.006
CKD (n, %)	2/24 (8.3)	13/175 (7.4)	0.873
HF (n, %)	0/24 (0.0)	1/175 (0.6)	0.711
AKI (n, %)	4/24 (16.7)	11/175 (6.3)	0.009
Non-survivors (n, %)	6/24 (25.0)	14/175 (8.0)	0.001
**Significant parameters**			
Male (n, %)	21/24 (87.5)	100/175 (57.1)	0.004
Specific weight	1.028 ± 0.011	1.021 ± 0.009	0.002
Urine glucose presence (n, %)	5/24 (20.8)	9/175 (5.1)	0.005
Urine protein presence (n, %)	20/24 (83.3)	80/175 (45.7)	0.001
Urine ketones presence (n, %)	3/24 (12.5)	5/175 (2.9)	0.024
Granular casts (cast/uL)	0.6 (1.2)	0.1 (0.4)	0.030
Hemoglobin (g/dL)	15.0 ± 1.6	14.2 ± 1.7	0.047
D-Dimer (ng/mL)	435 (576)	229 (216)	0.049
Serum creatinine (mg/dL)	0.95 (0.33)	0.81 (0.29)	0.032
Serum Sodium (mmol/L)	134 (4)	138 (4)	< 0.001
Serum Albumin (g/L)	33.5 ± 4.3	36.8 ± 3.8	< 0.001
LDH (U/L)	413 (225)	303 (147)	< 0.001
CRP (mg/dL)	11.89 (15.22)	6.41 (9.33)	0.027

Quantitative variables were described by mean ± standard deviation or median (IQR); DM, Diabetes; HT, Hypertension; CKD, Chronic kidney disease; HF, heart failure; ICU, Intensive Care Unit; AKI, Acute Kidney Injury; LDH, lactate dehydrogenase; CRP, C-reactive protein.

#### Acute kidney injury

**Figure 3:**
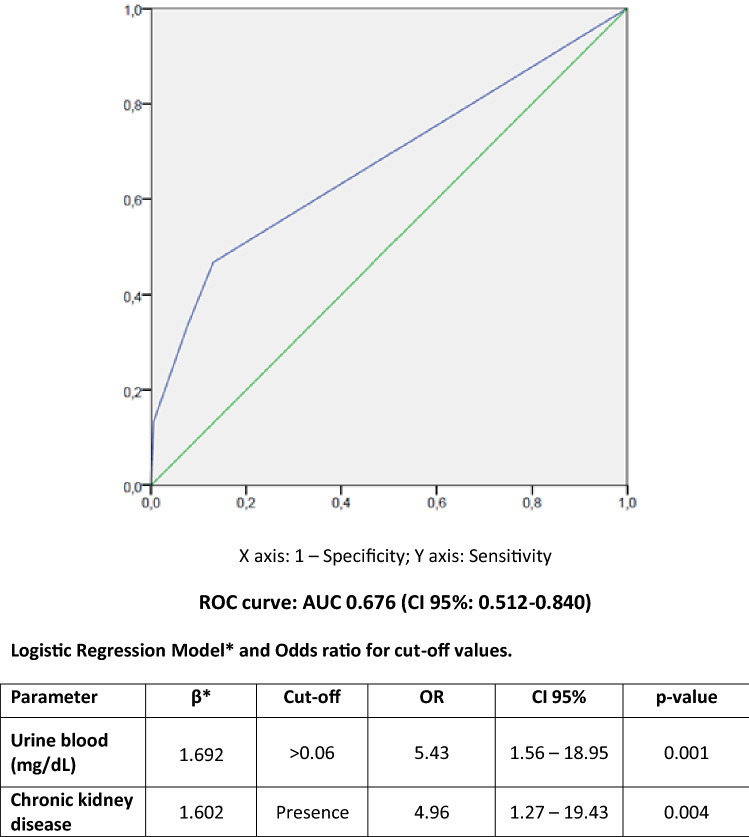
Cut-off values and ROC curve of urine blood and CKD for COVID-19 AKI prediction.

Fifteen patients developed AKI during the clinical episode, which represents 7.5% of the number of hospitalized patients. Clinical data and biochemical results for AKI and non-AKI patients are shown in Table 3. The ROC curve analyses performed on the different clinical variables and biomarkers revealed that the highest AUC was reached by a model including the presence of blood in urine and CKD, with an AUC of 0.676 (95% CI 0.512–0.840; *p* = 0.023). Different cut-off values of urine blood were tested in order to evaluate their ORs. Figure 3 summarizes cut-off values of urine blood for AKI prediction. Median time from urinalysis result to AKI development was 3.84 days (IQR: 6.09) and the specific features of these patients are summarized in Table 4.

**Table 3:** Clinical data and biochemical results for AKI and non-AKI COVID-19 patients.

Hospitalized patients	AKI	Non-AKI	*p*
N, %	15 (7.5%)	184 (92.5%)	< 0.001
Age (years)	64.1 ± 20.2	58.5 ± 15.9	0.250
DM (n, %)	4/15 (26.7)	23/184 (12.5)	0.014
HT (n, %)	11/15 (73.3)	64/184 (34.8)	< 0.001
Obesity (n, %)	5/15 (33.3)	14/184 (7.6)	0.001
CKD (n, %)	4/15 (26.7)	11/184 (6.0)	0.004
HF (n, %)	0/15 (0.0)	1/184 (0.5)	0.772
ICU (n, %)	4/15 (26.7)	20/184 (10.9)	0.004
Non-survivors (n, %)	5/15 (33.3)	15/184 (8.2)	< 0.001
**Significant parameters**			
Male (n, %)	11/15 (73.3)	110/184 (59.8)	0.049
Urine pH	5.5 (1.0)	6.0 (1.0)	0.017
Urine blood presence (n, %)	5/15 (33.3)	14/184 (7.6)	0.001
Urine protein presence (n, %)	14/15 (83.3)	128/184 (69.6)	0.030
Urine Nitrite positive (n, %)	2/15 (13.3)	5/184 (2.7)	0.032
Serum creatinine (mg/dL)	1.38 (0.90)	0.82 (0.27)	0.013
Serum Chloride (mmol/L)	106 (9)	104 (6)	0.017
Serum urate (mg/dL)	6.2 (2.3)	4.2 (2.0)	0.003

Quantitative variables were described by mean ± standard deviation or median (IQR); AKI, Acute Kidney Injury; DM, Diabetes; HT, Hypertension; CKD, Chronic kidney disease; HF, heart failure; ICU, Intensive Care Unit.

**Table 4:** Clinical data and biochemical results for AKI COVID-19 patients.

AKI patients N = 15	KDIGO Stage 1	KDIGO Stage 2	KDIGO Stage 3	*p*
N, %	10 (66.7)	2 (13.3)	3 (20.0)	< 0.001
Age (years)	59.8 (32.1)	84.3 (6.0)	71.3 (17.6)	0.002
Male (n, %)	7/10 (70.0)	2/2 (100)	2/3 (66.7)	0.009
DM (n, %)	2/10 (20.0)	2/2 (100)	0/3 (0)	0.002
HT (n, %)	7/10 (70.0)	2/2 (100)	2/3 (66.7)	0.009
Obesity (n, %)	4/10 (40.0)	0/2 (0)	1/3 (33.4)	0.001
CKD (n, %)	2/10 (20.0)	2/2 (100)	0/3 (0)	0.002
HF (n, %)	0/10 (0)	0/2 (0)	0/3 (0)	1.000
Episode time (days)	4 (5.5)	2 (0.5)	5 (8.6)	0.006
ICU (n, %)	2/10 (20.0)	0/2 (0)	2/3 (66.7)	0.001
ACET (n, %)	2/10 (20.0)	1/2 (50.0)	1/3 (33.4)	0.002
RRT (n, %)	1/10 (10.0)	0/2 (0)	1/3 (33.4)	0.009
TI (n, %)	2/10 (20.0)	0/2 (0)	2/3 (66.7)	0.001
Non-survivors (n, %)	2/10 (20.0)	0/2 (0)	3/3 (100)	< 0.001
Creatinine baseline (mg/dL)	1.22 (0.56)	3.10 (0.17)	0.91 (1.04)	0.001
Creatinine peak (mg/dL)	1.83 (0.68)	3.10 (0.17)	3.24 (0.92)	0.002
eGFR (mL/min per 1.73m^2^)	40.5 (16.0)	17.5 (0.5)	17.0 (5.0)	0.010

Quantitative variables were described by mean ± standard deviation or median (IQR); AKI, Acute Kidney Injury; DM, Diabetes; HT, Hypertension; CKD, Chronic kidney disease; HF, Heart failure; ICU, Intensive Care Unit; RRT, Renal replacement therapy; TI, Tracheal intubation; ACET, Angiotensin convertase enzyme inhibitors therapy; eGFR, Estimated glomerular filtration rate; KDIGO, Kidney Disease Improving Global Outcomes.

### Mortality

**Figure 4:**
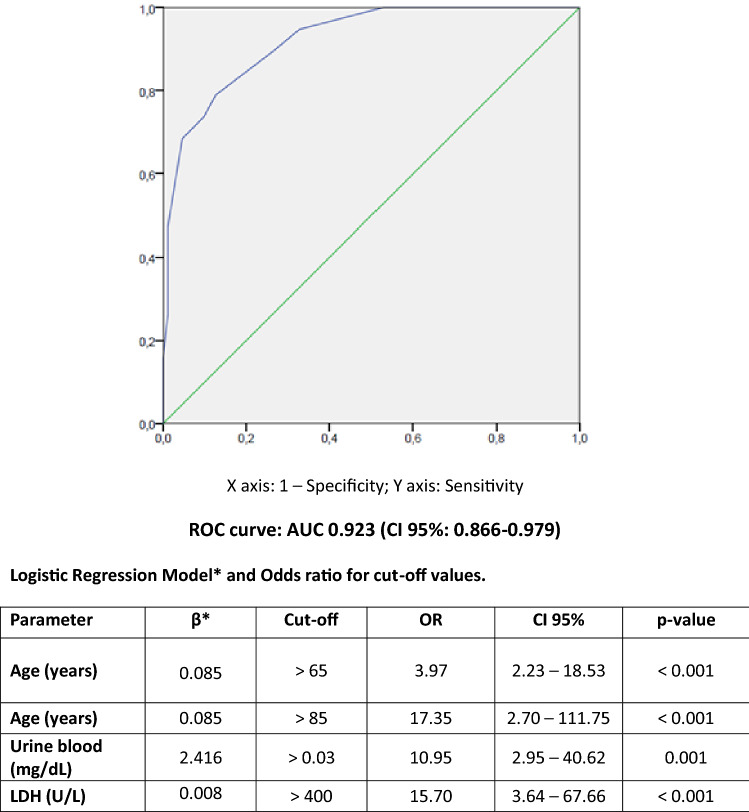
Cut-off values and ROC curve of age, urine blood and LDH activity in serum for COVID-19 mortality prediction.

Twenty patients died during hospital admission, which represents 10.1% of the number of hospitalized patients. Clinical data and biochemical results for survivors and non-survivors are shown in Table 5. The ROC curve analyses performed on the different clinical variables and biomarkers revealed that the highest AUC was reached by a model including age, presence of blood in urine and LDH levels in serum, with an AUC of 0.923 (95% CI 0.866–0.979; *p* < 0.001). Different cut-off values for age, urine blood and LDH levels in serum were tested in order to evaluate their ORs. Figure 4 summarizes the cut-off values of these variables for mortality prediction. Median time from urinalysis result to death was 8.04 days (IQR: 19.02).

**Table 5:** Clinical data and biochemical results for survivors and non-survivors COVID-19 patients.

Hospitalized patients	Non-Survivors	Survivors	*p*
N, %	20 (10.1%)	179 (89.9%)	< 0.001
Age (years)	73.0 ± 17.1	57.3 ± 15.4	0.029
DM (n, %)	0/20 (0.0)	27/179 (15.1)	< 0.001
HT (n, %)	12/20 (60.0)	63/179 (35.2)	< 0.001
Obesity (n, %)	2/20 (10.0)	17/179 (9.5)	0.810
CKD (n, %)	1/20 (5.0)	14/179 (7.8)	0.653
HF (n, %)	0/20 (0.0)	1/179 (0.6)	0.728
AKI (n, %)	5/20 (25.0)	10/179 (5.6)	0.002
ICU (n, %)	6/20 (30.0)	18/179 (10.1)	0.009
**Significant parameters**			
Male (n, %)	17/20 (85.0)	104/179 (58.1)	0.019
Urine blood presence (n, %)	11/20 (55.0)	24/179 (13.4)	< 0.001
Urine protein presence (n, %)	17/20 (85.0)	83/179 (46.4)	0.001
D-Dimer (ng/mL)	461 (770)	229 (224)	0.039
Serum Albumin (g/L)	32.6 ± 4.7	36.7 ± 3.7	0.003
LDH (U/L)	418 (186)	308 (143)	0.003
CK (U/L)	192 (163)	59 (77)	0.004
CRP (mg/dL)	14.71 (12.71)	6.32 (9.34)	0.007

Quantitative variables were described by mean ± standard deviation or median (IQR); DM, Diabetes; HT, Hypertension; CKD, Chronic kidney disease; HF, heart failure; ICU, Intensive Care Unit; AKI, Acute Kidney Injury; LDH, lactate dehydrogenase; CK, creatine kinase; CRP, C-reactive protein.

### Laboratory findings

In the study of serum and urine biomarkers in hospitalized patients, we observed interesting results in urine pH values, presence of blood and protein in the urine dipstick of those patients who develop AKI, as well as urate and chloride in blood. Regarding the need for admission to the ICU, a higher urine specific weight, ketones, glucose and protein were found in a greater percentage in individuals that presented a critical evolution than in those that didn’t, also highlighting a higher presence of pathological casts in the urinary sediments of these patients. In serum, increased concentrations of D-dimer, creatinine, LDH and CRP were observed, as well as a decrease in the albumin and sodium. Concerning mortality, the differential value of the urine dipstick with respect to the presence of blood and protein in a greater proportion in non-surviving patients was again noteworthy, as well as increased levels of D-dimer, LDH, CK and CRP, again observing a decrease in the level of serum albumin.

## Discussion

In this study, for patients with COVID-19, urine blood > 0.03 mg/dL and urine specific weight > 1.026 were shown to be associated with a high incidence of AKI, ICU admission and mortality. In addition, we found that patients with adverse clinical outcomes were predominantly older men with an age above 65 years, chronic kidney disease, lower levels of sodium and albumin in serum and an LDH activity > 400 U/L upon hospital admission, which correlated with the severity of the in-hospital episode compared to those hospitalized patients without these initial findings.

Biochemical parameters in urine can be used for UTI diagnosis, monitoring of kidney diseases and the follow-up of treatment effects, due to the easy sample collection, test automation and cost-effectiveness^19^. Despite that, only few studies reported on the correlation between urine biochemical parameters and the prediction of a critical evolution of COVID-19. Liu et al.^14^ found significant differences in the proportion of positive blood and protein in the urine dipstick between patients with COVID-19 and healthy controls. In addition, infected patients presented higher pH values and a decrease in urine specific weight. Regarding the severity of the COVID-19, the groups with the highest proportion of positive glucose and protein results were patients with the greatest severity; up to 60% of critical patients were positive for glucose in the urine dipstick and 50% for protein. These findings are consistent with those seen in our study regarding the group of patients admitted to the ICU versus those who did not, reporting a significant increase in the percentage of patients with positive values in urine dipstick glucose, protein, ketones and urine specific weight.

In a retrospective study, Pei G, et al.^18^ described that 75.4% of COVID-19 patients had renal involvement upon admission, 65.8% of which with proteinuria and 41.7% with hematuria. In their study as a whole, the presence of AKI was 4.7%, with a higher incidence during hospital stay than upon admission (86.4% vs 13.6%). Critically ill patients showed a high incidence of proteinuria and hematuria (85.7% and 69.6%, respectively), and also of AKI (42.9%). Mortality is proportional to the stage of AKI, reaching 90.9% in stage 3. However, overall mortality amounted 8.7% of admitted patients, and up to 52.8% of the critical patients. These results are consistent with those found in our study where the presence of proteinuria upon hospital admission was 62.8%, with a presence of 83.3% in the critical patient. The overall prevalence of AKI was 14.2%, and 26.7% of such patients required ICU admission. Mortality was higher among patients with AKI (33.3% vs. 8.2%), and the presence of blood in urine was a good predictor of in-hospital mortality in these patients.

Conversely, there are studies which question the relationship of CKD with the development of AKI and highlight that, in patients without CKD, the presence of COVID-19 does not increase the development of AKI, although this is a complication with a high mortality^20^. Our results confirm the presence of CKD as a predictor of the development of AKI in COVID-19 patients, with a risk 5 times higher than in COVID-19 patients without CKD. Moreover, the presence of AKI is related to a greater need for ICU admission and higher mortality in our population. These findings support the idea that the development of AKI in the COVID-19 patient is promoted by the patient's comorbidities, pharmacological treatments and the presence of a systemic inflammatory state as a consequence of viremia, and that this leads to a worse in-hospital evolution^21,22^. This observation is further supported by some case series reporting 19% of AKI in the population requiring ICU admission^23^.

The prediction of in-hospital mortality based on urinary parameters has already been evaluated by Bonetti G. et al.^24^ for COVID-19 patients attended at the Emergency Room. Urinary sediment was found to be a useful prognostic tool, as a higher percentage of in hospitalized non-survivors presented hyaline-granular casts and renal tubular cells, alongside with elevated serum creatinine and urea concentrations. In our study, no significant differences were seen in the presence of renal tubular cells between survivors and non-survivors, although assessed by another analytical technology^25^. Nevertheless, we highlighted the presence of a higher percentage of patients with pathological casts and higher serum creatinine values requiring ICU admission, which confirms the usefulness and convenience of an initial assessment of urine parameters (urine dipstick and sediment) and renal function.

Necropsy studies performed in non-survivors admitted to the ICU^26^ confirm that SARS-CoV-2 infection directly affects renal tissue and triggers the development of AKI, which is further exacerbated by systemic hypoxia, coagulation abnormalities and iatrogenic rhabdomyolysis. These observations would be reinforced by the fact that our study shows a higher incidence of AKI in ICU patients (16.7%), who also present an increase in D-Dimer, LDH and CRP levels, adding the finding of a higher CK activity in non-survivors. Other studies agree on the remark of a decreased serum albumin level upon hospital admission found in our study, as an independent factor for the development of stage 3 AKI and a fatal outcome^27^, adding value to early predictors of AKI by monitoring renal changes^28^. Our results shown in Table 4 confirm the differences between the different KDIGO stages of AKI, with a higher proportion of stage 3 patients with ICU admission, need for orotracheal intubation, renal replacement treatment, more days to renal function recovery and a high rate of mortality than those in stage 1 and 2.

In this regard, recent studies have described low serum albumin levels in young COVID-19 patients, along with elevated D-Dimer, LDH and the presence of proteinuria and obesity as predictors of critical disease progression^29^. Our findings are coincidental, since we reported a higher proportion of obese patients who develop AKI and require ICU admission. In addition, the presence of proteinuria, elevated D-dimer, LDH, CRP and decreased serum albumin are observed in those patients who are critical and non-survivors. Some authors have reported that the development of AKI during hospital episode combined with heart failure (HF) is related with high mortality rate^30^. In our study, HF does not appear to be associated with a worse evolution of the development of AKI. Altogether, our findings suggest that renal involvement with AKI presentation and the presence of urinary parameter alterations may add to respiratory involvement as prognostic factors of severity in COVID-19^31^.

The observed in-hospital mortality of 10.1% and the predictive model, including an age greater than 65 years, are in concordance with the findings reported in a recent meta-analysis, placing our data in an intermediate term between the Chinese cohorts with low mortality and the Spanish and US cohorts with in-hospital mortality rates of around 20%^32^. An influence of gender on adverse events has also been described^33^, the male gender presenting with a higher proportion of AKI, ICU admissions and mortality in our study. However, variables included in other studies^34^ such as lymphocyte and platelet counts and serum ferritin, were not included in our logistic regression models. We found that elevated LDH values above 400 U/L, together with an age above 65 years (more if it is greater than 85 years) and hematuria upon hospital admission have predictive capacity on the in-hospital mortality of COVID-19 patients. These findings are consistent with other Spanish and North American series^35–37^.

To the best of our knowledge, this study adds evidence to those who have included the prognostic role of urinary markers and their association with the three endpoints (AKI, need for ICU admission and in-hospital mortality) in small populations with COVID-19 individuals^38–40^.

This study has several *limitations*. First, during the COVID-19 outbreak, urine dipstick and sediment studies were scarcely requested, probably due to a low evidence in COVID-19 patients since only a few studies have attempted to reveal the prognostic role of urinary markers and their association with the renal injury in early pandemic times. These facts which might represent a selection bias in our retrospective study, especially with regard to the higher proportion of men included. Second, we did not detect SARS-CoV-2 in urine samples and no data was obtained from other biomarkers, biopsy studies or functional testing. Third, the prediction models presented need to be validated in prospective cohorts with control groups, especially since this is a small sample of AKI patients.

## Conclusions

This study shows the gender differences of the possible predictive variables in the hospital admission of COVID-19 patients. Three predictive models were included for AKI, need for ICU admission and mortality in which the independent variables derived from the early analysis of urine, together with an age above 65 years old, the presence of CKD and levels of serum markers such as albumin, sodium and LDH may represent a useful tool in the management of patients requiring hospital admission for a SARS-CoV-2 infection.

## Abbreviations

AKI: Acute kidney injury
ACE2: Angiotensin-converting enzyme receptor 2
AUC: Area under the curve
CKD: Chronic kidney disease
COVID-19: Coronavirus disease
CRP: C-reactive protein
CK: Creatinine phosphokinase
eGFR: Estimated glomerular filtration rate
HF: Heart failure
ICU: Intensive care unit
IQR: Interquartile ranges amplitude
KDIGO: Kidney Disease: Improving Global Outcomes
LDH: Lactate dehydrogenase
ROC: Receiver-operating characteristics
ARDS: Respiratory distress syndrome in the adult
RT-PCR: Reverse transcriptase-polymerase chain reaction
UTI: Urinary tract infection
